# Account of a Man Who Lives upon Large Quantities of Raw Flesh, in a Letter from Dr. Johnston, Commissioner of Sick and Wounded Seamen, to Dr. Blane

**Published:** 1800-03

**Authors:** J. Johnston


### *Account of a Man who lives upon large Quantities of raw Fleſh, in a Letter from* Dr. Johnston, Commiſſioner of Sick and Wounded Seamen, to Dr. Blane.

My dear Sir, *Somerſet Place, Oct. 28, 1799.*

HAVING in Auguſt and September laſt been engaged in a tour of public duty, for the purpoſe of ſelecting from among the priſoners of war ſuch men as, from their infirmities, were fit objects for being releaſed without equivalent, I heard, upon my arrival at Liverpool, an account of one of theſe priſoners being endowed with an appetite and digeſtion ſo far beyond any thing that had ever occured to me, either in my obſervation, reading, or by report, that I was deſirous of aſcertaining the particulars of it by occular proof, or undeniable teſtimony. Dr. Cochrane, Fellow of the College of Phyſicians of Edinburgh, and our medical agent at Liverpool, is fortunately a gentleman upon whoſe fidelity and accuracy I could perfectly depend; and I requeſted him to inſtitute an inquiry upon this ſubject, during my ſtay at that place. I incloſe you an atteſted copy of the reſult of this; and as it may probably appear to you, as it does to me, a document containing facts extremely intereſting, both in a natural and medical view, I will beg you to procure its inſertion in ſome reſpectable periodical work.

Some farther points of inquiry reſpecting this extraordinary perſon having occurred to me ſince my arrival in town, I ſent them in the form of queries to Doctor Cochrane, who has obligingly returned ſatisfactory anſwers. Theſe I ſend along with the above-mentioned atteſted ſtatement, to which I beg you to ſubjoin, ſuch reflections as may occur to you on this ſubject. I am,

My dear Sir,

Your moſt obedient humble ſervant,

J. JOHNSTON.

To Gilbert Blane, M. D. F. R. S. and one of the Commiſſioners of Sick and Wounded Seamen.

---

Charles Domery, a native of Benche, on the frontiers of Poland, aged 21, was brought to the priſon of Liverpool, in February 1799, having been a ſoldier in the French ſervice on board the Hoche, captured by the ſquadron under the command of Sir John B. Warren, off Ireland.

He is one of nine brothers, who, with their father, have been remarkable for the voracioufnefs of their appetites. They were all placed early in the army; and the peculiar craving for food with this young man, began at 13 years of age.

He was allowed two rations in the army, and by his earnings, or the indulgence of his comrades, procured an additional fupply.

When in the camp, if bread or meat were fcarce, he made up the deficiency by eating four or five pounds of grafs daily; and in one year, declares he devoured 174 cats, (not their fkins) dead or alive; and fays, he had feveral fevere conflicts in the act of deftroying them, by feeling the effects of their torments on his face and hands; fometimes he killed them before eating, but when very hungry, did not wait to perform this humane office.

Dogs and rats equally fuffered from his mercilefs jaws; and if much pinched by famine, the entrails of animals, indifcriminately, became his prey. The above facts are attefted by Picard, a refpectable man, who was his comrade in the fame regiment on board the Hoche, and is now prefent; and who affures me, he has often feen him feed on thofe animals.

When the fhip on board of which he was, had furrendered after an obftinate action, finding himfelf, as ufual, hungry, and nothing elfe in his way but a man's leg, which was fhot off, lying before him, he attacked it greedily, and was feeding heartily, when a failor fnatched it from him, and threw it overboard.

Since he came to this prifon, he has eat one dead cat and about twenty rats. But what he delights moft in, is raw meat, beef or mutton, of which, though plentifully fupplied by eating the rations of ten men daily,* he complains he has not the fame quantity, nor indulged in eating fo much as he ufed to do, when in France.

He often devours a bullock's liver, raw, three pounds of candles and a few pounds of raw beef, in one day, without tafting bread or vegetables, wafhing it down with water, if his allowance of beer is expended.

His fubfiftence at prefent, independent of his own rations, arifes from the generofity of the prifoners, who give him a fhare of their allowance. Nor is his ftomach confined to meat, for when in the hofpital, where fome of the patients refufing to take

* The French prifoners of war are at this time maintained at the expence of their own nation, and are each allowed the following daily ration: twenty-fix ounces of bread, half a pound of beef, half a pound of greens, two ounces of butter, or fix ounces of cheefe.

take their medicines, Domery had no objection to perform this for them; and his ftomach never rejected any thing, as he never vomits, whatever be the contents, or however large.

Wifhing fairly to try how much he actually could eat in one day; on the 7th of September, 1799, at four o'clock in the morning, he breakfafted on four pounds of raw cow's udder; at half paft nine, in prefence of Doctor Johnfton, commiffioner of fick and wounded feamen, Admiral Child and his fons, Mr. Fofter, agent for prifoners, and feveral refpectable gentlemen, he exhibited his powers as follows: There was fet before him five pounds of raw beef, and twelve tallow candles of a pound weight, and one bottle of porter; thefe he finifhed by half paft ten o'clock. At one o'clock, there was again put before him, five pounds of beef, and one pound of candles, with three bottles of porter; at which time he was locked up in the room, and fentries placed at the windows to prevent his throwing away any of his provifions. At two o'clock, when I again faw him with two friends, he had nearly finifhed the whole of the candles, and great part of the beef, but had neither evacuation by vomiting, ftool, or urine; his fkin was cool, and pulfe regular, and in good fpirits. At a quarter paft fix, when he was to be returned to his prifon, he had devoured the whole, and declared he could have eat more; but from the prifoners without, telling him, we wifhed to make fome experiment on him, he began to be alarmed. It is alfo to be obferved, that the day was hot, and not having his ufual exercife in the yard, it may be prefumed, he would have otherwife had a better appetite. On recapitulating the whole confumption of this day, it ftands thus:

| | | | |
|---|---|---|---|
| Raw cow's udder | | — | 4lb. |
| Raw beef | — | — | 10 |
| Candles | — | — | 2 |
| | Total | - | 16 lb. befide five bottles of porter. |

The eagernefs with which he attacks his beef when his ftomach is not gorged, refembles the voracity of a hungry wolf, tearing off large morfels with his teeth, rolling them about in his mouth, and fwallowing them with canine greedinefs. When his throat is dry from continued exercife, he lubricates it by ftripping the greafe off the candle between his teeth, which he generally finifhes at three mouthfuls, and wrapping the wick like a ball, ftring and all, fends it after at a fwallow. He can, when no choice is left, make fhift to dine on immenfe quantities of raw potatoes, or turnips; but, from choice, would never defire to tafte bread or vegetables.

He is in every respect healthy, his tongue clean, and his eyes lively.

After he went to the prison, he danced, smoaked his pipe, and drank a bottle of porter; and by four next morning, he awoke with his usual ravenous appetite, which he quieted by a few pounds of raw beef.

He is six feet three inches high, pale complexion, grey eyes, long brown hair, well made but thin, his countenance rather pleasant, and is good tempered.

The above is written from his own mouth, in the presence of, and attested by,

DESTAUBAN, French Surgeon.

LE FOURNIER, Steward of the Hospital.

REVET, Commissaire de la Prison.

LE FLEM, Garde du Magazin de l'Hospital.

PICARD, Soldat de la 1er Demi Brigade.

THOMAS COCHRANE, M. D. Inspector and Surgeon of the Prisons, and Agent, &c. for sick and wounded Seamen.

*Liverpool, Sept.* 9, 1799.

A true Copy,

JOHN BYNON, Clerk in the Office for sick and wounded Seamen.

### QUERIES and ANSWERS.

1. What is the quantity and quality of his fæces?

He goes to stool commonly morning and afternoon, in a smaller or larger quantity, according to the victuals he eats; but the fæces are by no means proportioned to the *ingesta*, and, indeed, seldom exceeding those of other men. They are always of the same consistence, generally hard, but of no extraordinary colour; and this last is the same, whether he eats vegetables or not with his animal food. They are not particularly offensive.

2. What are the circumstances of his sleep and perspiration?

He gets to bed about eight o'clock at night, immediately after which he begins to sweat, and that so profusely, as to be obliged to throw off his shirt. He feels extremely hot, and in an hour or two after goes to sleep, which lasts until one in the morning, after which he always feels himself hungry, even though he had lain down with a full stomach. He then eats bread or beef, or whatever provision he may have reserved through the day; and if he has none, he beguiles the time in smoking tobacco. About two o'clock he goes to sleep again, and awakes at five or six o'clock in the morning in a violent perspiration, with great heat. This quits him on getting up; and when he has laid in a fresh cargo of raw meat, (to use his own expression,) he feels his body in a good state. The perspiration, whether sensible or insensible, has no remarkable smell; indeed, much less than that of many persons who live on a very different different regimen. He ſweats while he is eating; and it is probably owing to this conſtant propenſity to exhalation from the ſurface of the body, that his ſkin is commonly found to be cool, as is ſtated in the narrative.

3. What is his heat by the thermometer?

I have often tried it, and found it to be of the ſtandard temperature of the human body. His pulſe is now eighty-four, full and regular.

4. Can this ravenous appetite be traced higher than his father?

He knows nothing of his anceſtors beyond his father. When he left the country, eleven years ago, his father was alive, aged about fifty, a tall, ſtout man, always healthy; and he can remember he was a great eater, but was too young to recollect the quantity, but that he eat his meat half boiled. He does not recollect that either himſelf, or his brothers, had any ailment, excepting the ſmall-pox, which ended favourably with them all. He was then an infant; his face is perfectly ſmooth.

5. Does he make much urine?

With his ordinary allowance of drink, not more than a quart in the day. He ſays, the ſmell is not more offenſive than that of other men. On board of the tranſport, coming from Ireland, he ſays, he drank his own urine as often as he voided it, for want of drink, and that he never vomited it.

6. Is his muſcular ſtrength greater or leſs than that of other men of his time of life?

Though his muſcles are pretty firm, I do not think they are ſo full or plump as thoſe of moſt other men. He has, however, by his own declaration, carried a load of three hundred weight of flour in France, and marched fourteen leagues in a day.

7. Is he dull or intelligent?

He can neither read nor write, but is very intelligent and converſable, and can give a diſtinct and conſiſtent anſwer to any queſtion put to him. I have put a variety at different times, and in different ſhapes, tending to throw all the light poſſible on his hiſtory, and never found that he varied, ſo that I am inclined to believe that he adheres to truth.

8. Under what circumſtances did this voracious diſpoſition firſt come on?

It came on at the age of thirteen, as has been already ſtated. He was then in the ſervice of Pruſſia, at the ſiege of Thionville; they were at that time much ſtraitened for proviſion, and as he found this did not ſuit him, he deſerted into the town. He was conducted to the French General, who preſented him with a large melon, which he devoured, rind and all, and then an immenſe quantity and variety of other ſpecies of food, to the great entertainment of that officer and his ſuite. From that time he he has preferred raw to dreſſed meat; and when he eats a moderate quantity of what has been either roaſted or boiled, he throws it up immediately. What is ſtated above, therefore, reſpecting his never vomiting, is not to be underſtood literally, but imports merely that thoſe things which are moſt nauſeous to others, had no effect upon his ſtomach.

9. Is there any thing remarkable regarding his venereal appetite or powers?

Upon inquiry I do not find there is any thing remarkable in this man, in theſe reſpects.

There is nothing farther to remark, but that ſince the atteſted narrative was drawn up, he has repeatedly indulged himſelf in the cruel repaſts there deſcribed, devouring the whole animal, except the ſkin, bones, and bowels; but this has been put a ſtop to, on account of the ſcandal which it juſtly excited.

In conſidering this caſe, it ſeems to afford ſome matters for reflection, which are not only objects of conſiderable novelty and curioſity, but intereſting and important, by throwing light on the proceſs by which the food is digeſted and diſpoſed of.

Monſtroſity and diſeaſe, whether in the ſtructure of parts, or in the functions and appetites, illuſtrate particular points of the animal œconomy, by exhibiting them in certain relations in which they are not met with in the common courſe of Nature. The power of the ſtomach in ſo quickly diſſolving, aſſimilating, and diſpoſing of the aliment in ordinary caſes, muſt ſtrike every reflecting perſon with wonder; but the hiſtory of this caſe affords a more palpable proof, and more clear conception of theſe proceſſes, juſt as objects of ſight become more ſenſible and ſtriking, when viewed by a magnifying glaſs, or when exhibited on a larger ſcale.

The facts here ſet forth, tend alſo to place in a ſtrong light, the great importance of the diſcharge by the ſkin, and to prove that it is by this outlet, more than by the bowels, that the recrementitious parts of the aliment are evacuated; that there is an admirable co-operation eſtabliſhed between the ſkin and the ſtomach, by means of that conſent of parts ſo obſervable, and ſo neceſſary, in the other functions of the animal œconomy; and, that the purpoſe of aliment is not merely to adminiſter to the growth and repair of the body, but by its bulk and peculiar ſtimulus to maintain the play of the organs eſſential to life.

